# Glucagonoma syndrome with serous oligocystic adenoma: A rare case report

**DOI:** 10.1097/MD.0000000000008448

**Published:** 2017-10-27

**Authors:** Yun Gao, Chun Wang, Yunyi Gao, Huijiao Chen, Bing Peng, Weixia Chen, Xingwu Ran

**Affiliations:** a Diabetic Foot Center, Department of Endocrinology and Metabolism; b Department of Pathology; c Department of Pancreatic Surgery; d Department of Radiology, West China Hospital, Sichuan University, Chengdu, Sichuan, People's Republic of China.

**Keywords:** diabetes mellitus, glucagonoma, necrolytic migratory erythema, pancreas, serous oligocystic adenoma

## Abstract

**Rationale:**

Glucagonoma and pancreatic serous oligocystic adenoma (SOA) are rare neuroendocrine and exocrine tumors of the pancreas, respectively. The coexistence of glucagonoma syndrome (GS) and SOA is a rare clinical condition and has not yet been reported. Additionally, necrolytic migratory erythema (NME), a hallmark clinical sign of GS, is often misdiagnosed as other skin lesions by clinicians due to their lack of related knowledge, which delays diagnosis of GS and thus exacerbates the prognosis.

**Patient concerns:**

A 50-year-old male patient was admitted to our department because he presented with diabetes mellitus and a recurrent ulcerated skin rash. Prior to the accurate diagnosis, the skin manifestation was considered to be diabetic dermopathy.

**Diagnoses:**

The patient's fasting serum glucagon level was up to 871.5 pg/mL. A biopsy of the pancreatic tumor revealed a pancreatic neuroendocrine tumor, and immunoperoxidase staining revealed glucagon-positive cells. In addition, the histological examination of the pancreatic cystic lesions showed typical features of SOA.

**Interventions:**

The patient received a pancreaticoduodenal resection and radiofrequency ablation for the hepatic nodular lesion.

**Outcomes:**

One week after surgery, the glucagon concentration decreased to near normal levels. The cutaneous lesions spontaneously resolved within 4 weeks after surgery.

**Lessons:**

Because almost all glucagonomas are malignant or have malignant potential, their early recognition and correct diagnosis are very important for a better prognosis, especially in cases with NME as the only manifestation during the development of glucagonomas. It is therefore imperative that clinicians recognize NME early to make an accurate diagnosis.

## 1 Introduction

Pancreatic neuroendocrine tumors (PNETs), commonly referred to as islet cell tumors, are rare neoplasms. Glucagonoma is a type of PNET derived from islet alpha cells and has an estimated incidence of approximately 1 in 20 million people per year.^[1]^ The overproduction of glucagon may result in “glucagonoma syndrome (GS),” which includes necrolytic migratory erythema (NME), diabetes mellitus, anemia, weight loss, and other features.

Pancreatic serous cystic neoplasm, including serous microcystic adenoma, serous oligocystic adenoma (SOA), and serous cystic carcinoma, account for 1% to 2% of all exocrine pancreatic tumors.^[2]^ SOA, which represents approximately 10% of serous cystic neoplasms, is a relatively rare tumor lesion of the pancreas.^[3]^ Although serous cystadenomas are often reported to coexist with other pancreatic neoplasms,^[4]^ the coexistence of SOA and a rare pancreatic endocrine neoplasm is an uncommon clinical condition and has not yet been reported.

We herein present a case of GS combined with SOA arising from the pancreas, which is a rare combination of pancreatic endocrine and exocrine tumors.

## 2 Case report

Our study was approved by the Institutional Ethic Committee of West China Hospital, Sichuan University. Written informed consent was obtained from the patient before data collection.

**Figure 1:**
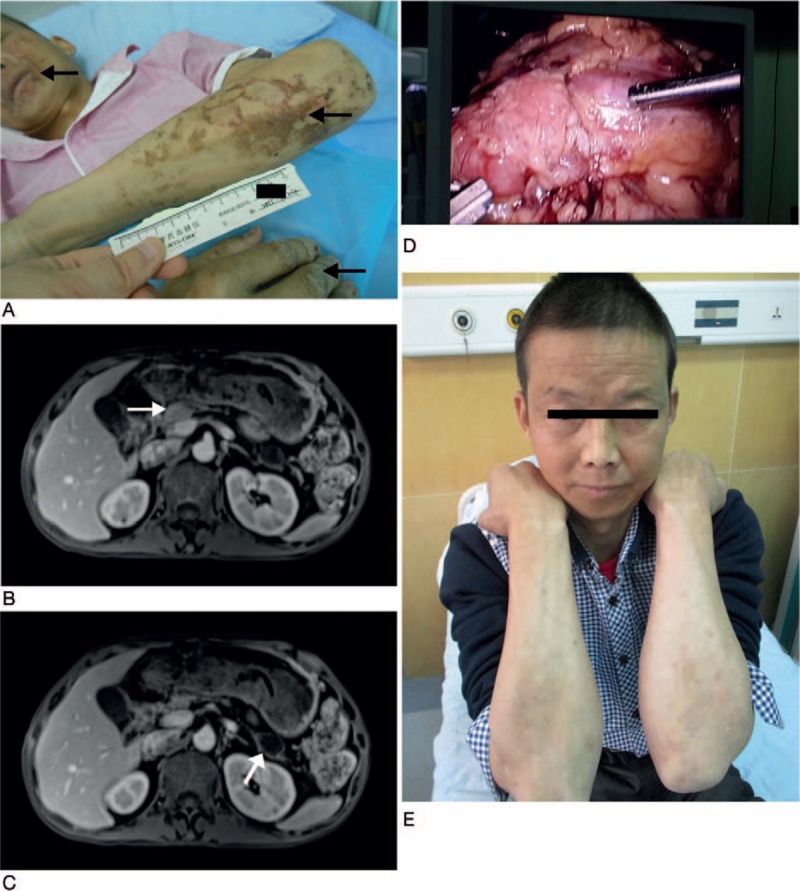
(A) Ulcerated skin rash on the upper extremities and perioral skin (black arrow). (B, C) Abdominal enhanced MRI reveals a suspected mass lesion in the head (white arrow) and a cystic lesion in the tail of the pancreas (white arrow), respectively. (D) The intraoperative tumor image is shown. (E) Within 4 wk after surgery, the skin manifestations spontaneously resolved.

In August 2015, a 50-year-old male patient of Chinese Han nationality was admitted to the hospital's Endocrinology Department because of an ulcerated skin rash that recurred for 11 years and hyperglycemia for 8 years. The patient had diffuse and recurrent erythematous and erosive lesions that were primarily on his extremities, perioral region, gluteal sulcus, and perineum (Fig. 1A). The rash had been worsening despite specialized wound care. Eight years prior to admission, the patient was evaluated in the general clinic of a local hospital due to polyuria, polydipsia, and weight loss and was then diagnosed with diabetes based on a random glucose test result of 26 mmol/L. After insulin administration for half a year, the patient switched to oral hypoglycemic agents. His blood glucose was originally under control but became unregulated over time. He also suffered from diabetic ketoacidosis 4 months prior. Additionally, the patient noted concurrent glossitis, balanitis, and alternating diarrhea and constipation. The patient had no family history of these complications. On physical examination, the patient had stable vital signs but was physically thin (BMI: 17.6 kg/m^2^) with reddish-crusted lesions mainly in the perioral region and distal extremities.

Laboratory tests revealed normocytic normochromic anemia (hemoglobin, 87 g/L), hypoalbuminemia (33.9 g/L, normal value 40.0–50.0 g/L), and hyperglycemia (fasting glucose, 8.01 mmol/L). The level of glycosylated hemoglobin was elevated to 7.5%. The level of fasting chromogranin A, which is a serum marker for neuroendocrine neoplasia, was elevated (625.7 ng/mL, normal range <94 ng/mL). The level of fasting serum glucagon was also elevated (871.5 pg/mL, normal value <200 pg/mL) and was accompanied by an elevated glucose level of 8.01 mmol/L.

**Figure 2:**
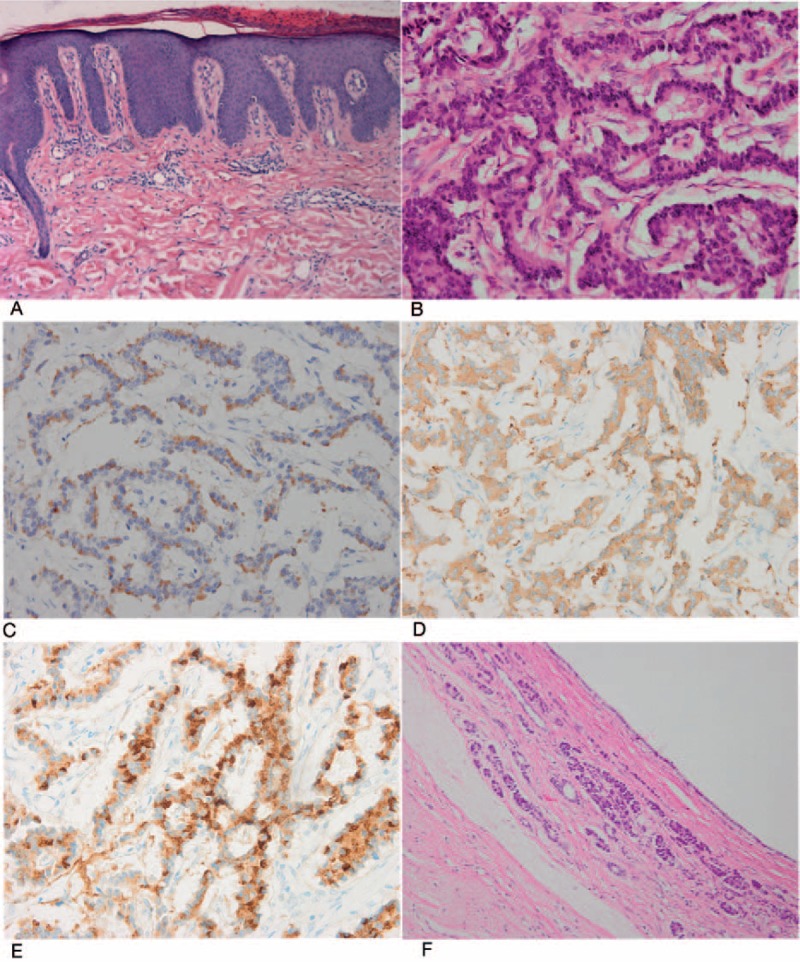
(A) Histopathological sections demonstrate acanthosis, diffuse parakeratotic hyperkeratosis, necrosis in the epidermis, and neutrophilic infiltration (H&E, original magnification × 400). (B) Microscopic examination of the pancreatic head neoplasm with a solid pattern (H&E, original magnification × 400). (C, D, E) Immunohistochemical staining shows positive findings for glucagon, synaptophysin, and chromogranin A, respectively. (F) Microscopic examination reveals a serous cystadenoma composed of an irregular-shaped cyst lined by cuboidal and flattened epithelial cells with bland nuclei and clear cytoplasm.

A biopsy of the skin lesion showed acanthosis and diffuse parakeratotic hyperkeratosis in the epidermis. Neutrophil infiltration and focal hemorrhage were present in the cornified layers of the skin, and perivascular lymphocytic infiltration was observed in the upper dermis (Fig. 2A). Based on these findings, a diagnosis of NME was considered. An abdominal MRI revealed a suspected mass in the head of the pancreas (Fig. 1B) and several cystic lesions in the atrophic body and tail of the pancreas (Fig. 1C). In addition, there was a small solid nodular lesion in the right posterior lobe of the liver.

On hospital day 25, the patient was transferred to the Department of Pancreatic Surgery and then underwent a pancreaticoduodenal resection with the da Vinci Surgical System (Fig. 1D) and radiofrequency ablation for the hepatic nodular lesion. Intraoperatively, 2 solid masses (3 and 2 cm in diameter) were identified in the head of the pancreas, and 2 cystic lesions were identified in the body and tail of the pancreas, which were also approximately 3 cm and 2 cm in diameter, respectively. The pathological analysis of the solid masses (Fig. 2B) revealed a PNET. Immunohistochemical staining was positive for glucagon (Fig. 2C), synaptophysin (Fig. 2D), and chromogranin A (Fig. 2E), whereas the staining was negative for insulin, gastrin, and somatostatin. The findings were consistent with a diagnosis of a glucagonoma. In addition, histological examination of the cystic lesions showed typical features of SOA (Fig. 2F).

The association of a glucagonoma tumor with NME confirmed the diagnosis of GS. One week after the surgery, the patient's cutaneous lesions almost disappeared. Simultaneously, the glucagon concentration decreased to near normal levels (248.8 pg/mL) and was accompanied by a fasting glucose level of 8.73 mmol/L. Moreover, the total daily insulin dose was reduced from 60 units before the operation to 26 units after the glucagonoma was removed. Within 4 weeks after surgery, the skin manifestations spontaneously resolved (Fig. 1E). At 3 months after surgery, the total daily insulin dose was continually reduced to approximately 10 units, and the HbA1c level at follow-up was 6.6%. However, the patient was prone to recurrent episodes of hypoglycemia. There was still no sign of cutaneous or systemic disease.

## 3 Discussion

Glucagonoma is an extremely rare neuroendocrine tumor, comprising only 2% to 5% of all islet cell tumors.^[5,6]^ NME is a hallmark clinical sign of GS and is present in approximately 70% of GS patients.^[1]^ Although the exact pathogenesis remains unclear, the development of NME is considered to be due to the direct action of glucagon in inducing skin necrolysis, hypoaminoacidemia or a deficiency of essential fatty acids or zinc.^[7]^ As NME may be the only manifestation during the development of glucagonomas that is malignant, its early recognition and correct diagnosis is very important for a better prognosis. Unfortunately, clinicians often misdiagnose NME as eczema, psoriasis, or contact dermatitis on account of their lack in related knowledge.^[1]^ In the present case, the patient was thought to have eczema and was treated for eczema for almost 3 years before being diagnosed with diabetes mellitus. Regardless of whether the combination of NME and diabetes developed, community doctors did not recognize the disease, which was misdiagnosed for almost 11 years.

Diabetes mellitus is another common finding. Case studies suggest that 80% to 94% of patients with GS gradually develop diabetes mellitus during the course of the disease.^[8]^ Previous studies reported that the blood glucose level of GS patients was mildly elevated and well controlled with dietary measures.^[9]^ By the time of diagnosis, a Mayo Clinic report suggested that only 38% of GS patients presented with diabetes mellitus, which generally developed before NME in patients with both conditions.^[10]^ However, our patient presented with thirst, polyuria, polydipsia, and rapid weight loss (lost almost 5 kg within a week) before diabetes was established. At the onset of the patient's diagnosis of diabetes, he had to use insulin injections to control blood glucose because his fasting capillary blood glucose was up to >26 mmol/L. The development of diabetes in this patient seemed to be rapid. We speculate that the clinical findings may be associated with his pancreatic atrophy, of which the exact cause is still unclear. The other basic clinical features of the disease are weight loss, anemia, diarrhea, thromboembolism, and neuropsychiatric symptoms. Our patient did have obvious weight loss, normocytic anemia, diarrhea, calf muscular venous thrombosis, and agitation.

The symptoms and signs constituting GS reveal the presence of a glucagonoma. However, the diagnosis of GS still requires an elevated serum glucagon level (usually 500–1000 pg/mL) and imaging confirming a pancreatic tumor. Almost 75% of glucagonoma was located in the tail of the pancreas, and most patients already present with metastatic disease, for which the most common site is the liver.^[11]^ However, the tumor in our patient was located in the head of the pancreas, which is uncommon for this disease. There was a node in the liver that might also be considered liver metastasis.

In our case, it was most surprising that GS coexisted with a rare exocrine pancreatic tumor, SOA. Previous studies reported that serous cystadenomas often coexisted with other pancreatic neoplasms.^[4]^ To our knowledge, however, this is the first report of GS coexisting with SOA. As glucagonoma may present as part of the multiple endocrine neoplasia (MEN) diseases, we originally suspected the presence of a MEN in the patient; however, except for the 2 rare neoplasms, we did not find clinical or imaging results related to other neoplasms such as pituitary adenoma, thyroid tumor, or adrenal adenoma. Although the coexistence of pancreatic endocrine tumor with serous cystadenoma has also been described in certain cases,^[12]^ the endocrine tumors reported in these studies were accidentally discovered based on postoperative histological pathology rather than clinical signs of hormone hyperproduction. So far, the coexistence of GS with typical clinical manifestations and SOA has not yet been reported.

Presently, surgical therapy is still the most effective treatment and should be considered as an optimal choice for patients with GS, regardless of whether most patients have metastasis at diagnosis.^[13]^ Patients with metastatic disease can still experience a relatively prolonged survival (>20 years) because this tumor type is slow-growing.^[6]^ In addition, treatment by resection of the tumor produces rapid clinical resolution, as noted in our patient. For patients with contraindications to surgery, long-acting somatostatin analogs and systemic chemotherapy, which are considered as palliative treatments, are also alternative treatment strategies and may provide good control of symptoms.^[14–16]^

In summary, we presented a very rare case of coexisting GS and SOA. The fact that the patient remained without a correct diagnosis for a long time indicates again that it is very important for clinicians to be familiar with NME to diagnose GS early. Once the combination of NME and diabetes developed, the initial clinical impression of GS should be considered.
